# Correction to: Inhibition of ghrelin-induced feeding in rats by pretreatment with a novel dual orexin receptor antagonist

**DOI:** 10.1007/s12576-019-00665-w

**Published:** 2019-02-14

**Authors:** Mariko So, Hirofumi Hashimoto, Reiko Saito, Yukiyo Yamamoto, Yasuhito Motojima, Hiromichi Ueno, Satomi Sonoda, Mitsuhiro Yoshimura, Takashi Maruyama, Koichi Kusuhara, Yoichi Ueta

**Affiliations:** 1 Department of Health and Nutritional Care, Faculty of Medical Science, University of East Asia, Shimonoseki, 751-0807 Japan; 2 Department of Physiology, School of Medicine, University of Occupational and Environmental Health, 1-1 Iseigaoka, Yahatanishi-ku, Kitakyushu, 807-8555 Japan; 3 Department of Pediatrics, School of Medicine, University of Occupational and Environmental Health, Kitakyushu, 807-8555 Japan

## Correction to: J Physiol Sci DOI 10.1007/s12576-016-0517-5

The article Inhibition of ghrelin-induced feeding in rats by pretreatment with a novel dual orexin receptor antagonist, written by Mariko So, Hirofumi Hashimoto, Reiko Saito, Yukiyo Yamamoto, Yasuhito Motojima, Hiromichi Ueno, Satomi Sonoda, Mitsuhiro Yoshimura, Takashi Maruyama, Koichi Kusuhara, Yoichi Ueta, was originally published electronically on the publisher’s internet portal (currently SpringerLink) on 4 January 2017 without open access.

With the author(s)’ decision to opt for Open Choice the copyright of the article changed on 15 February 2019 to © The Author(s) [2019] and the article is forthwith distributed under the terms of the Creative Commons Attribution 4.0 International License (http://creativecommons.org/licenses/by/4.0/), which permits use, duplication, adaptation, distribution and reproduction in any medium or format, as long as you give appropriate credit to the original author(s) and the source, provide a link to the Creative Commons license and indicate if changes were made.

